# Unilateral congenital giant megaureter with renal dysplasia compressing contralateral ureter and causing bilateral hydronephrosis: a case report and literature review

**DOI:** 10.1186/s12894-016-0125-y

**Published:** 2016-02-09

**Authors:** Mingming Yu, Geng Ma, Zheng Ge, Rugang Lu, Yongji Deng, Yunfei Guo

**Affiliations:** Department of Urology, Nanjing Children’s Hospital Affiliated to Nanjing Medical University, Nanjing, Jiangsu 210029 China

**Keywords:** Congenital giant megaureter, Hydronephrosis, Renal dysplasia, Congenital megaureter

## Abstract

**Background:**

Congenital giant megaureter (CGM) is uncommon in the pediatric population. The major clinical presentations are marked protruberances and abdominal cysts.

**Case presentation:**

We reported a case of CGM with almost the whole left ureter dilation accompanied with a 1 cm stricture at the entrance of the bladder and renal dysplasia, immediately compressing the contralateral ureter and causing bilateral hydronephrosis for the first time. At one-stage of the operation, a left nephrostomy with a right ureterolysis were performed, and a poor left kidney function was found. Then, the left kidney and ureter were cut off by nephroureterectomy at the second-stage. Eventually, the follow-up showed that the patient recovered well by abdominal ultrasound.

**Conclusion:**

Based on the findings of these reported literatures, CGM is rare. The physical and imaging examinations are essential for the diagnosis of CGM, and the appropriate treatment methods should be performed based on patients’ specific condition.

## Background

Congenital giant megaureter (CGM) is an extremely rare condition, which is defined as “the lumen of a ureter is congenitally, focally and segmentally dilated to more than 10 times of the normal diameter, in presence of normal bladder volume and function [1].” The first CGM was reported by Chaterjee SK [2] in 1964. Since then, a small number of patients with CGM have been reported and a PubMed search yielded less than 10 published case reports to date.

Herein, we reported an entirely dilated CGM accompanied with 1 cm stricture at the entrance of the bladder and renal dysplasia, thereby compressing the contralateral ureter and causing bilateral hydronephrosis in a 3-year-old boy. In addition, we reviewed the epidemiology, pathogenesis, diagnosis and therapies of this rare condition by analyzing all previously reported cases.

## Case presentation

**Fig. 1:**
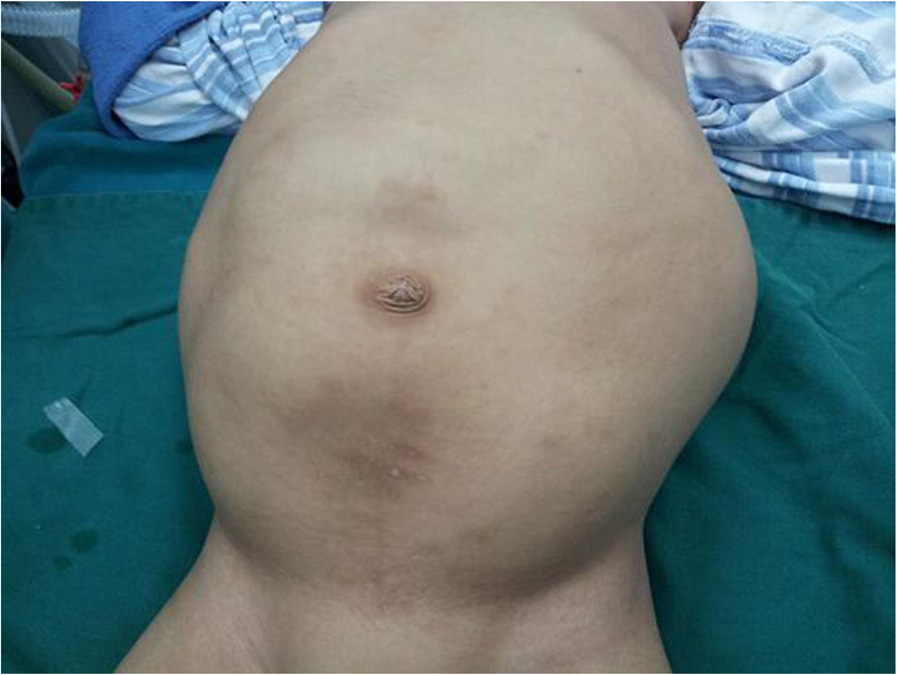
Physical examination shows a big abdominal circumference

**Fig. 2:**
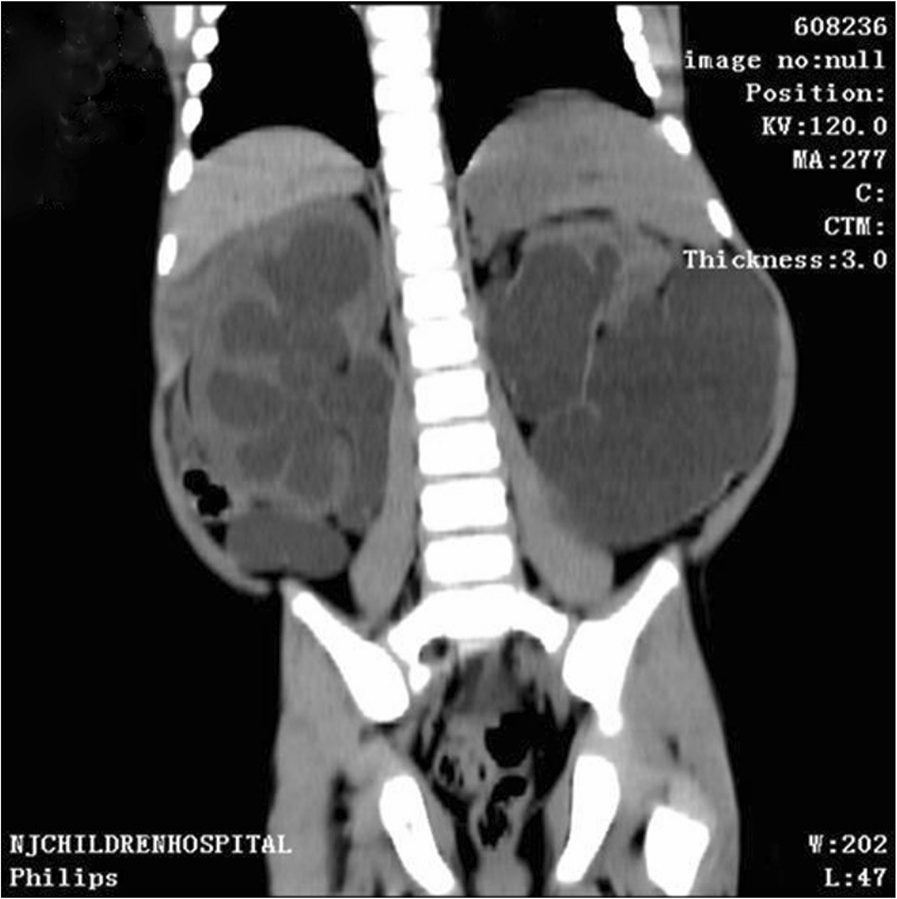
Abdominal computed tomography shows a giant ureter on the left side and right hydronephrosis with the whole right ureter dilatation

**Fig. 3:**
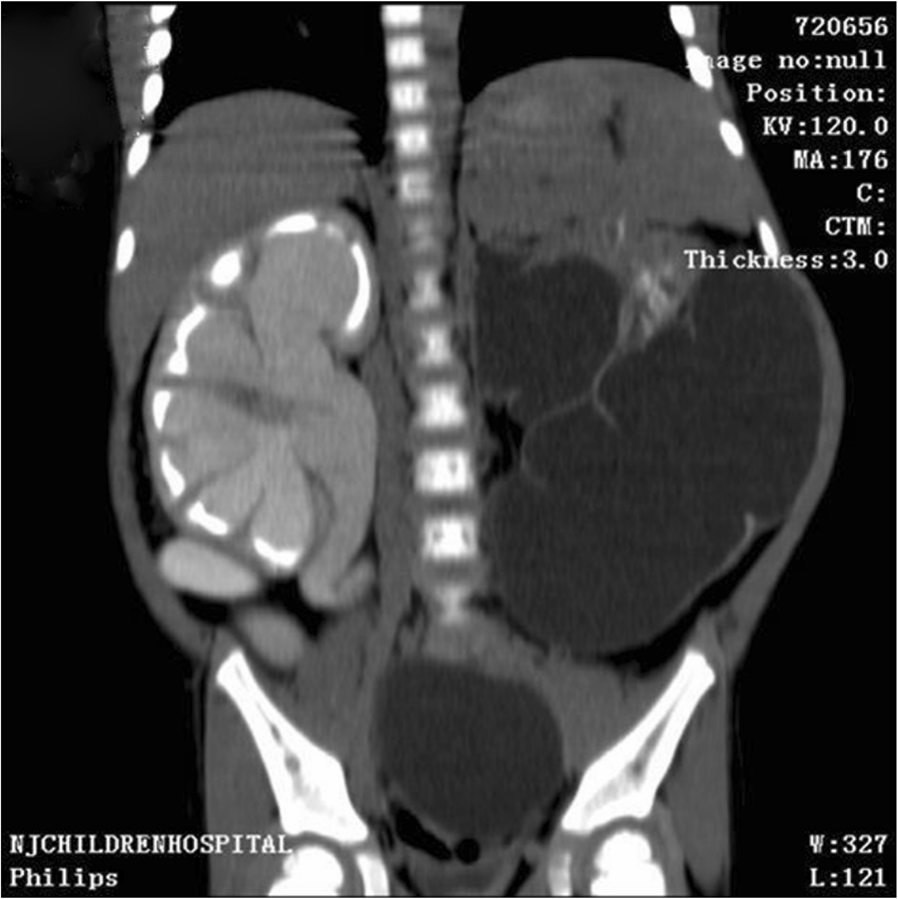
Contrast-enhanced computed tomography scan shows renal dysplasia with giant ureter on the left side and right hydronephrosis with the whole right ureter dilatation

A 3-year-old boy presented to our hospital with a big abdominal circumference (Fig. 1) since he was born. He had no history of urinary tract infection or flank pain. The abdominal examination showed a defined cystic abdominal mass with a smooth surface measuring 15 × 10 cm. The abdominal ultrasound revealed a separated acoustic dark area on the left abdomen and bilateral hydronephrosis with upper ureter dilatation on the right abdomen. Similarly, abdominal computed tomography (CT) scan demonstrated a giant ureter on the left side and right hydronephrosis with the whole dilatation of right ureter (Fig. 2). Contrast-enhanced CT scan further showed renal dysplasia with a giant ureter (Fig. 3). In addition, a dynamic diethylene triamine pentaacetic acid (DPTA) radionuclide renogram showed no function in the left glomeruli and compensatory increase in the right glomeruli. On cystoscopy, the left ureteric orifice could not be found. Based on these examinations, a diagnosis of left CGM causing a malfunction of the left kidney and bilateral hydronephrosis was made.

**Fig. 4:**
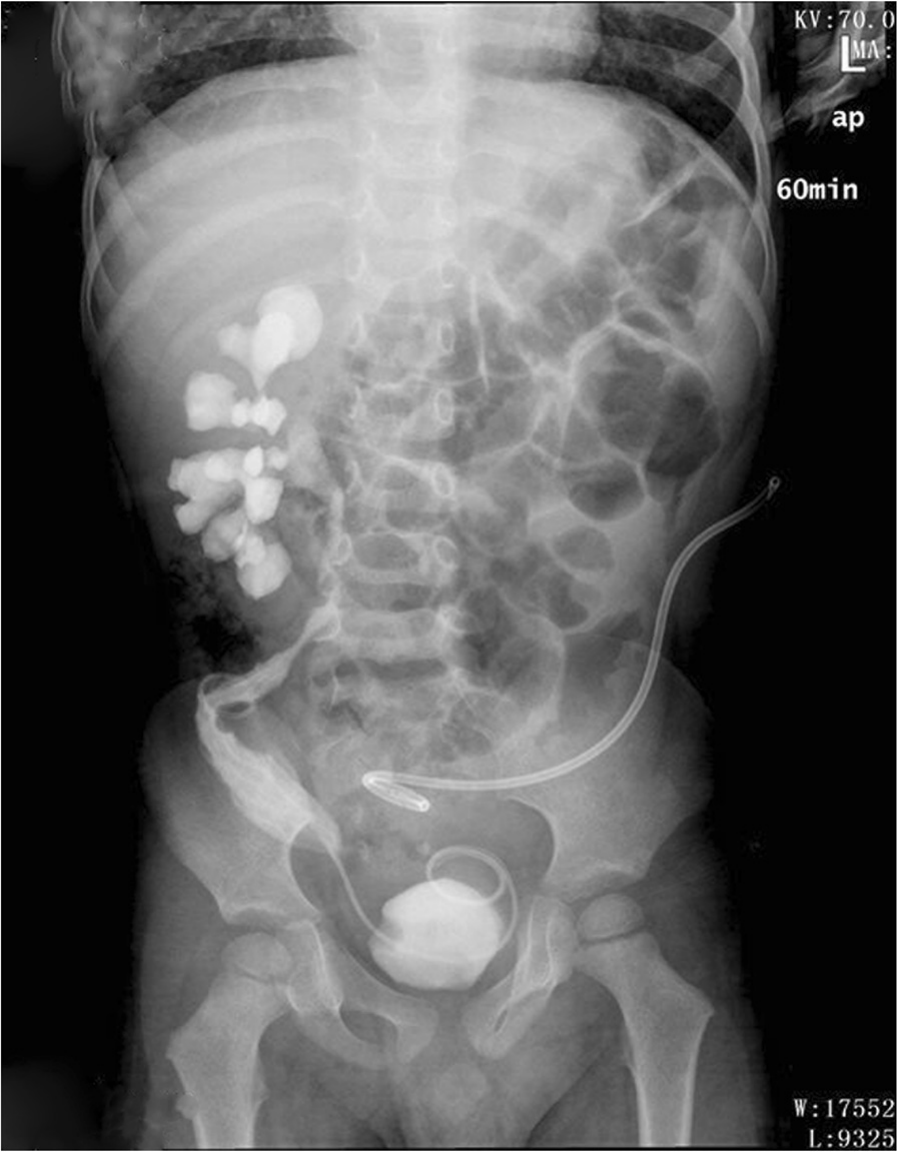
Intravenous pyelography reveals no images of the left kidney and ureter, and also shows the compressed right ureter

**Fig. 5:**
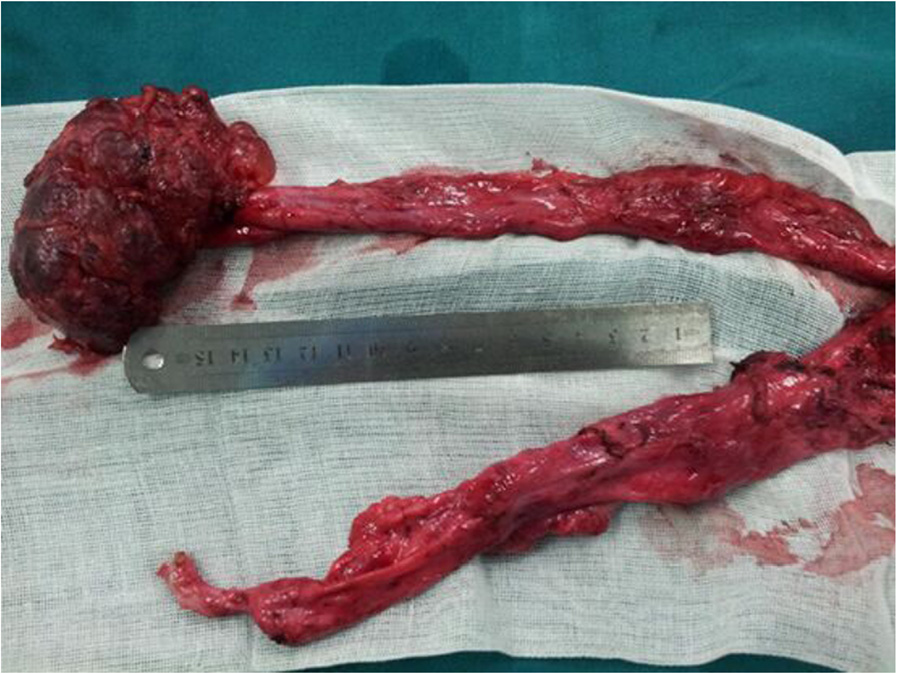
The dilated ureter is about 40 cm and we can observe the small left kidney with many vesicles on the surface and the stricture in the distal segment of the dilated ureter

**Fig. 6:**
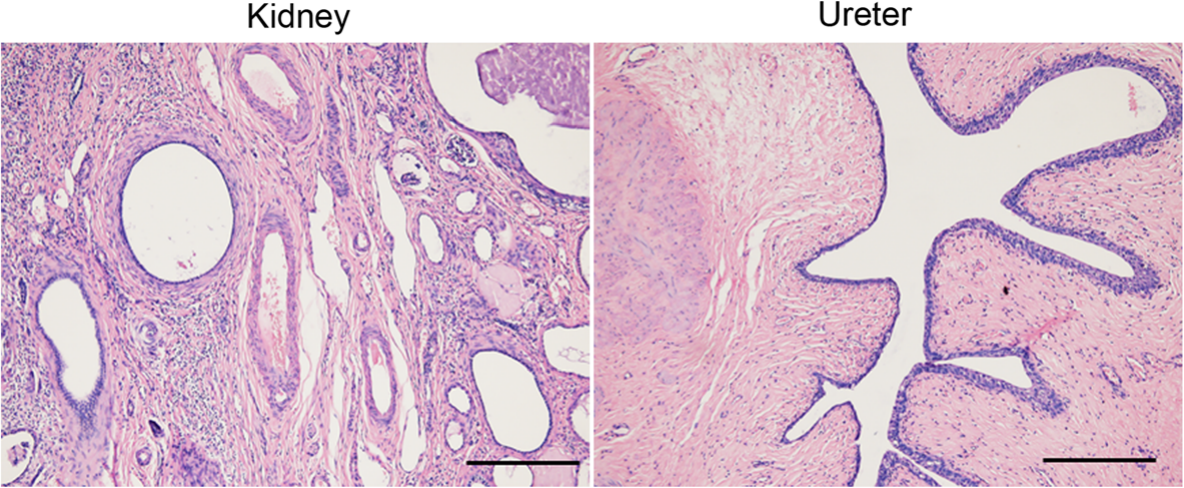
The postoperative pathologic examination shows the multicystic dysplastic kidney and ureter with fibroplasia. Bar = 100 μm

**Fig. 7:**
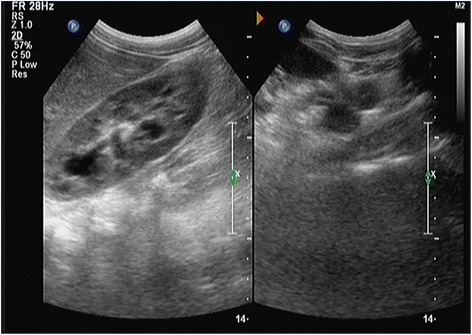
The follow-up abdominal ultrasound shows remarkably reduced right hydronephrosis

At one-stage of the operation, the giant left ureter and the right ureter dilated about 5 cm from the entrance of the bladder (the submucosal segment of the ureter) were found in the deep right bladder. So we considered that the right ureter was compressed by the giant left ureter, and then a left nephrostomy with a right ureterolysis were performed. After the first operation, the liquid outflowing from the single J tube was about 10 mL per day. After the first operation for 19 days, a dynamic DPTA radionuclide renogram was performed again and revealed a serious decline in the function of left kidney. In addition, an intravenous pyelography showed no images of the left kidney and ureter (Fig. 4). These results indicated a poor left kidney function and we considered that the left kidney could not be kept any more. As a result, a second-stage operation was performed thirty days after the first operation. During the operation, we could see a dysplastic left kidney and an almost entirely dilated left ureter with only 1 cm stricture at the entrance of the bladder, then nephroureterectomy was performed through cutting off the left kidney and ureter close to the bladder (Fig. 5). The postoperative pathologic examination showed that the left kidney and ureter were similar to multicystic dysplastic kidney (Fig. 6). The patient recovered well and remarkably reduced right hydronephrosis was found by the follow-up abdominal ultrasound (Fig. 7). The patient was observed to be asymptomatic after 2 years of follow-up.

## Discussion

CGM is extremely rare in the pediatric population. To the best of our knowledge, only 27 cases have been reported in the English literatures [1, 3–8] (Table 1). Among 27 patients with CGM, the ratio of women/men was approximately 1:1, indicating that there was no sex difference in CGM, while congenital megaureter occurred more often in men [9]. The megaureter often began from birth to pre-school age. There were 2 cases with CGM from birth, 6 cases before one year old, 8 cases from one to three years old, and 10 cases from four to eight years old. The oldest patient reported was 15 years old. Unlike congenital megaureter which might be observed bilaterally in about 20 % cases [10], all of the 27 patients with CGM were unilateral with 14 megaureter on the left side and 13 megaureter on the right side.

**Table 1:** Case reports on congenital giant megaureter

Author, year	The number of cases	Age	Treatments	Follow-up	Outcomes
Huang [1], 1987	21	Ranged from 2 months to 8 years	Nephrectomy/heminephrectomy and resection of the giant megaureter	-	Nineteen patients: free of urinary symptoms; One girl: died
					One boy: poorly recovered
Chiesa et al. [3], 2001	1	1-day-old	Nephroureterectomy	Four years	Uneventful with normal right renal function, a normal bladder and urethra
Ramaswamy et al. [4], 1995	1	2-year-old	Ureteroureterostomy.	-	Uneventful
Saurabh et al. [5], 2010	1	7-year-old	Surgical exploration was planned	-	-
Khattar et al. [6], 2009	1	15-year-old	Nephroureterectomy.	One year	Recovered well
Goto et al. [7], 2010	1	1-day-old	Ureteroureterostomy	Eighteen months	Experienced two febrile urinary tract infection, and no obstruction in the right upper urinary tract
Annigeri et al. [8], 2012	1	20-day-old	Nephroureterectomy.	Nine months	Uneventful

Currently, the pathogenesis of CGM or congenital megaureter is considered to be related to the expression of transforming growth factor β which might lead to a lack of post-natal muscle dysplasia [11, 12]. In the earlier study, Mackinnon et al. [13] put forward a theory that a lack of longitudinal muscle in the distal ureter led to the functional obstruction, which was accepted by many scholars. Then, Notley et al. [14] found the normal nerves distribution and collagen fiber hyperplasia in the muscular layer of the megaureters by the electron microscopy, which was considered as the major reason of the megaureter. In addition, Tokunaka et al. [15, 16] described a small subgroup of megaureters with muscle dysplasia which affected the dilated part of the ureter, and muscle dysplasia was thought as the primary cause leading to the dilatation. In recent years, most scholars believed that multiple factors contributed to the congenital megaureter.

The diagnosis of CGM was usually based on the history, the physical examinations and imaging examinations. In the present case, the diagnosis of CGM with the left giant ureter immediately compressing the contralateral ureter and causing bilateral hydronephrosis was made according to the physical examination and the imaging examination mainly including the abdominal ultrasound, the abdominal CT and the intravenous pyelography. Abdominal ultrasound was a basic methods to reveal the rough morphology of the kidney and ureter. Intravenous urography was the major diagnostic method, which could show the extent of the dilated ureter and renal pelvis, as well as the peristalsis and morphology of the ureter, thereby estimating the renal function. Besides, magnetic resonance urography (MRU) combined with urography could clearly reveal the features of megaureter, including the extent of the dilated ureter and renal pelvis, as well as the location of the narrow segment [17]. Therefore, MRU might be a good choice for infant patients.

The treatment of congenital megaureter is controversial. Upadhyay et al. [18] proposed an early surgical therapy, while Chertin et al. [19] suggested a conservative treatment temporarily for most patients. Compared with congenital megaureter, the treatment of CGM is specific. Ureteroureterostomy following the excision of the dilated segment or ureteral re-implantation was effective for patients with segmental dilation and the preserved renal function; however, for the patients with the whole dilated ureter and poor renal function, nephroureterectomy might be a good choice [3–5]. Noteworthily, during nephroureterectomy, it was essential to protect the compressed contralateral ureter and kidney [6].

## Conclusion

This study described an unilateral CGM with renal dysplasia compressing contralateral ureter and causing bilateral hydronephrosis in a 3-year-old boy. Based on the findings of these reported literatures, CGM is rare. The physical examinations and imaging examinations are essential for the precise diagnosis of CGM, and the appropriate treatment methods such as nephrostomy, ureterolysis, ureteroureterostomy and nephroureterectomy, should be performed based on patients’ specific condition. However, further studies on the pathogenesis of CGM are recommended.

## Consent

Written informed consent was obtained from the patient’s parents for publication of this case report and any accompanying images.

## Abbreviations

CGM: Congenital giant megaureter
CT: Computed tomography
DPTA: Diethylene triamine pentaacetic acid
DRF: Differential renal function
MRU: Magnetic resonance urography
